# Mechanical characterization of base analogue modified nucleic acids by force spectroscopy†

**DOI:** 10.1039/d1cp01985f

**Published:** 2021-06-22

**Authors:** Vinoth Sundar Rajan, Xavier Viader-Godoy, Yii-Lih Lin, Uttama Dutta, Felix Ritort, Fredrik Westerlund, L. Marcus Wilhelmsson

**Affiliations:** Department of Chemistry and Chemical Engineering, Chalmers University of Technology Sweden marcus.wilhelmsson@chalmers.se; Department of Biology and Biological Engineering, Chalmers University of Technology Sweden fredrik.westerlund@chalmers.se; Small Biosystems Lab, Condensed Matter Physics Department, Universitat de Barcelona, C/Marti i Franques 1 Barcelona 08028 Spain

## Abstract

We use mechanical unfolding of single DNA hairpins with modified bases to accurately assess intra- and intermolecular forces in nucleic acids. As expected, the modification stabilizes the hybridized hairpin, but we also observe intriguing stacking interactions in the unfolded hairpin. Our study highlights the benefit of using base-modified nucleic acids in force-spectroscopy.

## Abstract 2

Investigating the forces in nucleic acids using single base-pair level modifications and optical tweezers.

## Graphical abstract

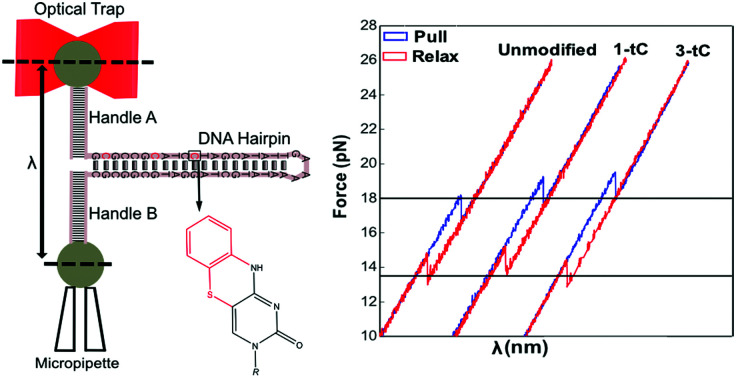

Understanding DNA structure and stability is vital as it changes during fundamental biological processes, such as replication, transcription, repair and chromatin organization.^1,2^ The stability of nucleic acids mainly depends on base-pair stacking and hydrogen bonding.^3–5^ Thus, the stability of nucleic acids can be altered and characterized by substituting the natural bases with modified nucleobases that affect these interactions. Synthetically prepared modified nucleobases are generally designed to enable complementary base-pairing and to give minimal perturbation to the native structure.^6–8^ An interesting subgroup of these is the fluorescent base analogues, which have emerged as a tool to investigate structure, dynamics and functions of nucleic acids and their interactions, mostly in bulk experiments.^9–11^ The fluorescent tricyclic cytosine analogue tC (Fig. 1A) forms three hydrogen bonds with guanine, but is expected to slightly change the stacking interactions due to its extended aromatic system compared to normal cytosine.^12–14^ Hence, tC incorporation at one or more places in a sequence provides a way to, with base-pair resolution, investigate how such small changes affect the properties of nucleic acids.

**Fig. 1:**
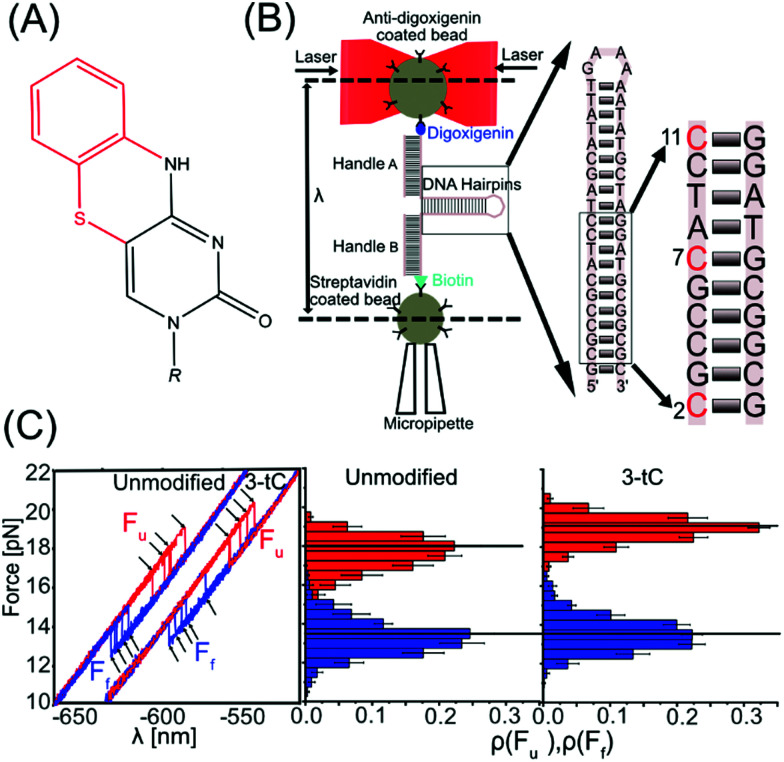
Experimental setup and stability of DNA hairpins. (A) Structure of tC; red represents additional part compared to cytosine. (B) Schematic representation of the optical tweezers. Inset shows the hairpins used with tC modification(s) in different positions in red. (C) Five force–distance cycles for an unmodified and a three-tC hairpin (3-tC) with unfolding (red) and folding curves (blue) and the corresponding rupture force histograms. The black horizontal lines indicate the median force.

Nucleic acid properties are conventionally studied using bulk techniques, like thermal melting, calorimetry and various UV/Vis-absorption techniques.^3,15^ In recent years, force spectroscopy has emerged as a technique to study the properties of nucleic acids at the single-molecule level.^16–22^ As an example, nearest neighbour base-pair energies in DNA have been measured with 0.1 kcal mol^−1^ accuracy.^23,24^ Moreover, base modifications and mismatches were recently studied using force spectroscopy.^25–29^ The literature on using synthetic base analogues in single-molecule experiments is very limited,^30–32^ but we envision that it will be a powerful tool for studying structure and dynamics of nucleic acids. Here, we combine optical tweezers and thermal melting to study how structural stability, (un)folding thermodynamics and intermolecular forces are affected by substitution of C with tC.

We used optical tweezers to manipulate single DNA hairpins by pulling on their ends *via* duplex handles, attached to two micron-sized beads, and applied forces in the pN range with nm resolution. One bead was optically trapped, while the other was attached to a micropipette by suction. The DNA hairpin was (un)folded by moving the optical trap that changed the distance between the position of the trap and the bead in the micropipette (*λ*), thereby applying a mechanical force (Fig. 1B). The DNA hairpins were synthesized by incorporating tCs at various positions (Table S1, ESI†). The unmodified DNA hairpin is shown in Fig. 1B (inset) and tC-modifications (at positions 2, 7, and 11), corresponding to 1-tC (11), 2-tC (7, 11) and 3-tC (2, 7, 11), are shown in red. In the abasic site hairpin (Abasic) an abasic site replaces G opposite to tC in position 11. As observed in the force–distance curves (FDC, Fig. 1C and Fig. S1, ESI†), the DNA hairpins unfolded (*F*_u_, red) and folded (*F*_f_, blue) *via* a single-step transition. *F*_u_ and *F*_f_ were extracted using custom-made MATLAB codes and the force histograms are shown in Fig. 1C and Fig. S1, ESI.†

Circular dichroism in the region between 200 and 300 nm shows a significant similarity among all hairpins, confirming that the tC-modifications do not perturb the native structure of the DNA hairpins (Fig. S2A, ESI†). Fig. 2 and Table 1 show how *F*_u_ and *F*_f_ changed upon incorporation of tC (at 1 M NaCl). *F*_u_ increases significantly (statistical analysis in ESI,† Tables S2 and S3) with tC-incorporations (1-tC, 2-tC, 3-tC) compared to the unmodified hairpin (Table S2, ESI†); one tC insertion resulted in a ∼1 pN increase and 3-tC was the most stable hairpin. An increasing trend in *F*_u_ with the number of tCs was also observed from the linear fit (Fig. S3A, ESI†) and supported by Pearson's correlation coefficient:^33^ 0.91, and *r* value: 0.85 (though the *F*_u_ values are within the error bars). Additionally, the increase in *F*_u_ between 1-tC and 3-tC was found to be significant (Table S2, ESI†) indicating that the stabilizing effect was qualitatively additive with the number of tCs. Contrastingly, the change in *F*_f_ was negligible with tC incorporation (Fig. 2 and Table S3, ESI†). The Abasic hairpin showed a significant decrease in both *F*_u_ and *F*_f_. Whereas the increase in *F*_u_ when incorporating tC could be explained by enhanced base-stacking propensity of the extended tC base compared to C, the negligible change in *F*_f_ is intriguing. We hypothesize that this is because the major contribution in *F*_f_ is the number of hydrogen bonds, which is preserved with tC substitution. The free energy (Δ*G*) was calculated from the FDC using Crook's fluctuation theorem (Fig. S4, ESI†).^34,35^ The calculated Δ*G* for the unmodified hairpin (57(1) *k*_B_*T*) agreed well with the mfold-predicted^36,37^ Δ*G* (56*k*_B_*T*). tC incorporation increased the free energy up to ∼3*k*_B_*T* (Fig. 2), rendering more stable hairpins, with the 3-tC hairpin showing the highest stability. The stabilization was again qualitatively additive (statistical analysis in Fig. S3B, ESI†). The Abasic hairpin, as expected, was destabilized by ∼4*k*_B_*T*.

**Fig. 2:**
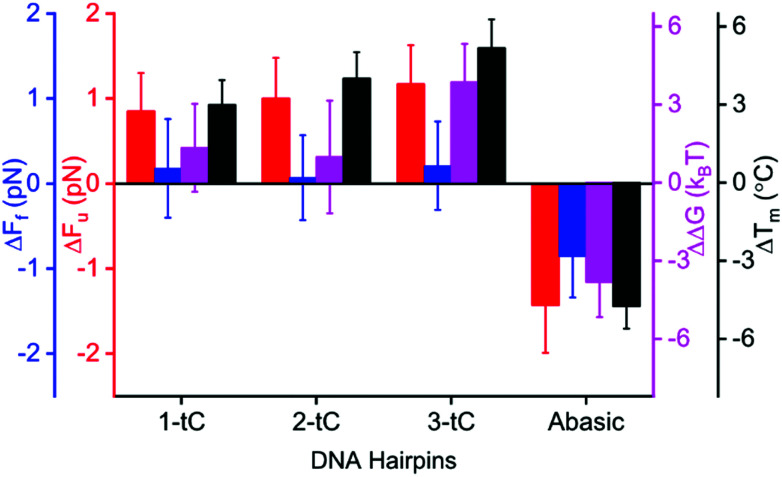
Mechanical and thermal stabilities of tC-modified DNA hairpins. Changes in unfolding (Δ*F*_u_, red) and folding force (Δ*F*_f_, blue), free energy (ΔΔ*G*, purple) from mechanical (1 M NaCl) and melting temperature (Δ*T*_m_, black, 5 mM NaCl) measurements comparing tC-modified and unmodified hairpins.

**Table 1:** Parameters from optical tweezers and bulk thermal measurements on hairpins at 1 M and 5 mM NaCl

DNA hairpin	*n* a	*F*_u_ b (pN)	*F*_f_ b (pN)	Δ*G*^c^ (*k*_B_*T*)	*T*_m_ d (°C)
Unmodified	854	18.0 (4)	13.4 (4)	57 (1)	69.7 (7)
1-tC	840	18.8 (3)	13.6 (4)	59 (1)	72.7 (6)
2-tC	990	19.0 (3)	13.5 (4)	58 (2)	73.7 (7)
3-tC	790	19.1 (3)	13.7 (3)	61 (1)	76.4 (7)^e^
Abasic	643	16.5 (4)	12.6 (3)	53 (1)	65.0 (5)
2-tC(opp)	679	18.8 (4)	13.7 (4)	58 (1)	71.0 (5)
2-tC(stack)	792	17.9 (4)	12.6 (4)	52 (1)	75.8 (6)

a Number of (un)folding cycles (*n*) obtained from different bead pairs (*N*), 8 < *N* < 10 in optical tweezers experiment (1 M NaCl).

b Unfolding (*F*_u_) and folding (*F*_f_) force are reported as median ± standard error of median.

c Free energy (Δ*G*) calculated using Crook's fluctuation theorem (Fig. S4, ESI) and mean ± standard error of mean.

d Melting temperature from UV-absorbance thermal experiments reported as mean ± standard deviation from two experiments.

e The *T*_m_ measurements for 3-tC was performed using a different instrument and its Δ*T*_m_ (see Fig. 2) is calculated using the *T*_m_ value for the unmodified hairpin in the same instrument (71.2 °C, *i.e.* 1.5 °C above the measurement on unmodified in other instrument, 69.7 °C). The values reported in parentheses refer to the error associated with the last significant figure.

The increased stability observed in the unfolding experiments agreed with thermal melting experiment at 5 mM NaCl (Material and methods, Fig. 2 and Fig. S2B, ESI†). The melting temperature (*T*_m_) increased gradually and was 5.2 °C higher for the 3-tC (*α*-curve fit of melting indicate a virtually clean two-state model, Fig. S2C, ESI†) compared to the unmodified hairpin. The Abasic hairpin was, as expected, less stable than the unmodified hairpin (−4.7 °C). Overall, the hairpin showed a similar trend in the mechanical and thermal measurements, where the stabilization with number of tC-incorporations was qualitatively additive. Again, we suggest that this stabilization is predominantly due to increased base-stacking interactions.

Next, we were interested in investigating how an extended base analogue like tC interacts with itself and the consequences that has on hairpin stability. Hence, hairpins with tCs on opposite strands (2-tC(opp), modified at positions 7 and 39) or with two tCs stacking on top of each other in the same strand (2-tC(stack), modified at position 10 and 11) were synthesized (Fig. 3A and Fig. S1, ESI†). The increase in *F*_u_ of the 2-tC(opp) was similar to the 1-tC and 2-tC hairpins (*vide supra*) while *F*_f_ again was very similar to the unmodified hairpin (Tables S2 and S3, ESI†). The increase in Δ*T*_m_ for 2-tC(opp) was smaller as compared to other tC-modified constructs. This is likely due to the contributions from neighboring bases, as reported before.^38^

**Fig. 3:**
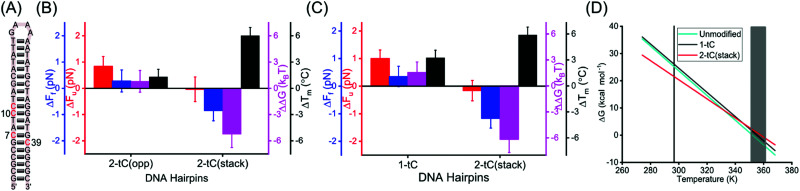
Mechanical and thermal stabilities of tC-modified hairpins. (A) DNA hairpins 2-tC(opp) and 2-tC(stack) with tC modifications. (B and C) Changes in unfolding force (Δ*F*_u_, red), folding force (Δ*F*_f_, blue), free energy (ΔΔ*G*, purple) at 1 M NaCl and melting temperature (Δ*T*_m_, black) at 5 mM NaCl for 2-tC(opp) and 2-tC(stack) (B) and both experiments at 50 mM NaCl for 1-tC and 2-tC(stack) (C) compared to the unmodified hairpin. (D) Calculated change in free energy (Δ*G*) with temperature for unmodified, 1-tC and 2-tC(stack) hairpins. Vertical line represents the temperature where optical tweezers experiments were conducted and shaded region corresponds to the melting temperatures of the hairpins.

Interestingly, 2-tC(stack) behaves very differently; *F*_u_ was similar compared to the unmodified hairpin, whereas *F*_f_ was significantly lower (Tables S2 and S3, ESI†). This observation agrees with the free energies calculated (Table 1). Interestingly, the destabilization of the 2-tC(stack) was not observed in the *T*_m_ measurements, where 2-tC(stack) showed an increase in *T*_m_ (+6.1 °C) compared to the unmodified hairpin; the largest increase for any of the hairpins studied.

The different behavior of 2-tC(stack) in optical tweezers and melting experiments compared to the control is interesting to understand in detail. An important discrepancy between these experiments is that they were performed at different NaCl concentrations (1 M and 5 mM, respectively). Therefore, we repeated both experiments at an intermediate ionic strength (50 mM) for the unmodified, 1-tC and 2-tC(stack) hairpins (Fig. 3C, Table 2 and Fig. S5, ESI†). The results were similar to Fig. 2 and 3B for both the modified hairpins, demonstrating that differences were not an effect of the ionic strength.

**Table 2:** Parameters from optical tweezers and bulk thermal measurements on hairpins at 50 mM NaCl

DNA hairpins	*n* a	*F*_u_ b (pN)	*F*_f_ b (pN)	Δ*G*^c^ (*k*_B_*T*)	*T*_m_ d (°C)	−Δ*H*^c^^,^^e^ (kcal mol^−1^)	−Δ*S*^c^^,^^e^ (cal K^−1^ mol^−1^)
Unmodified	651	14.6 (3)	9.6 (4)	43 (1)	79.0 (5)	160 (5)	454 (17)
1-tC	844	15.6 (3)	9.9 (4)	44 (1)	82.2 (7)	158 (5)	444 (15)
2-tC(stack)	812	14.4 (4)	8.4 (3)	37 (1)	84.9 (8)	126 (5)	351 (16)

a Number of un(folding) cycles (*n*) obtained from different bead pairs (*N*), 8 < *N* < 10.

b Unfolding (*F*_u_) and folding (*F*_f_) force reported as median ± error of median.

c Calculated free energy (Δ*G*), enthalpy (Δ*H*) and entropy (Δ*S*) represented as mean ± standard error of mean.

d Melting temperature (*T*_m_) from UV-absorbance thermal experiments reported as mean ± standard deviation from two experiments.

e Enthalpy and entropy calculated by combining Δ*G* (mechanical measurements) and *T*_m_ (thermal measurements) as described in Materials and methods. The values reported in the parentheses refer to the error associated with the last significant figure.

The data at 50 mM NaCl was used to determine the enthalpy (Δ*H*) and entropy (Δ*S*) by combining Δ*G* from the mechanical measurements at 23.5 °C and *T*_m_ from the melting experiments (where Δ*G* = 0), using Δ*G* = Δ*H* − *T*Δ*S* (Materials and methods, ESI†). The values for the unmodified hairpin (−Δ*H*: 160(5) kcal mol^−1^, −Δ*S*: 454(17) cal K^−1^ mol^−1^) agreed with mfold-predicted values (−Δ*H*: 164 kcal mol^−1^, −Δ*S*: 464 cal K^−1^ mol^−1^). Both Δ*H* and Δ*S* were similar (within errors) for 1-tC and the unmodified hairpin but decreased by ∼20% for 2-tC(stack) (Table 2). The computed Δ*H* and Δ*S* were used to interpolate Δ*G* between the two experimental temperatures (Fig. 3D). 1-tC Showed a slightly higher Δ*G* compared to the unmodified hairpin over the entire temperature range. On the other hand, Δ*G* for 2-tC(stack) showed a significantly different temperature dependence, where Δ*G* was higher at the hairpin-melting temperature range and lower at the temperature where the mechanical measurements were conducted. This explains why 2-tC(stack) behaved differently in the mechanical measurements compared to the melting experiments. A possible explanation for this large difference is stabilization of the single-stranded form of the 2-tC(stack) hairpin by stacking of the two tC bases at low temperatures. Such stacking likely gives rise to a combination of single-stranded semi-folded structures^39,40^ with (1) locally less conformational freedom (S lower) and, (2) increased π-stacking interactions between bases (H lower) at, and in close proximity to, the two tCs, which in turn decreases the free energy gain for duplex formation. The enthalpy and entropy for the other tC-modified hairpins can be computed indirectly by correcting Δ*G* from 1 M to 50 mM or 5 mM and combining with *T*_m_ data at 50 mM or 5 mM (Tables S4, S5 and Fig. S6, ESI†). These results confirm the conclusions at 50 mM NaCl.

To summarize our findings, (a) isolated tC bases increase the thermal and mechanical stability of DNA hairpins and the stability depends on the number of tCs, and (b) stacking of two adjacents tCs stabilizes the single-stranded form relative to adjacent natural bases. The combination of thermal and mechanical measurements was necessary to resolve the underlying reason for this and identified a different temperature dependence of Δ*G* for the hairpin with stacked tCs. Combining optical tweezers with base analogues allows convenient, and with base-pair precision, local modification of the stability, structure and dynamics of nucleic acids. This strategy can be extended to understand how local modification of properties affect important biological processes, such as replication, transcription, and repair, as well as studies of processes involving RNA and RNA–protein interactions. Moreover, by combining base analogues and force microscopy we have pointed out the direction towards interbase single-molecule FRET for nucleic acids in optical tweezers experiments with fluorescence readout. We have previously shown this to be a promising technique to study structure and dynamics of nucleic acids in bulk^41–43^ and with the improved photophysics of fluorescent base analogues recently reported, for example pA and 2CNqA,^32,44,45^ we suggest that interbase-FRET in optical tweezers will soon become an important tool in single-molecule studies.

FR, FW and MW designed the project. VSR performed all experiments. XV-G assisted with the single molecule experiments and analysis of that data, supervised by FR. VSR, Y-LL and UD developed analysis software. VSR, FW and MW wrote the paper. All authors commented on the paper. FW and MW contributed equally and coordinated the project.

This work was supported by a grant to FW and MW from the EI-Nano at Chalmers University of Technology. FW acknowledges funding from the European Research Council in the form of a Consolidator Grant (nanoDNArepair, no. 866238). We acknowledge Niklas Bosaeus for technical assistance with the instrument and for providing initial data analysis scripts. XV-G and FR acknowledge support from the Spanish Research Council Grant PID2019-1111486B-100. FR acknowledges support from ICREA Academia Prize 2018.

## Conflicts of interest

The authors declare no competing financial interests.

## Supplementary Material

CP-023-D1CP01985F-s001
