# Supplementary material for: Mechanical characterization of base analogue modified nucleic acids by force spectroscopy
Source: Phys Chem Chem Phys. 2021 Jun 22;23(26):14151–5. doi: 10.1039/d1cp01985f (PMC8261857; doi:10.1039/d1cp01985f)
Supplement: CP-023-D1CP01985F-s001 [file CP-023-D1CP01985F-s001.pdf]

Electronic Supplementary Material (ESI) for Physical Chemistry Chemical Physics.
This journal is © the Owner Societies 2021

Supplementary Information

Mechanical characterization of base analogues modified nucleic acids by force spectroscopy

Vinoth SUNDAR RAJAN[a,b], Xavier VIADER-GODOY[c], Yii-Lih LIN[b] , Uttama DUTTA[b], Felix RITORT[c], Fredrik WESTERLUND[b*] and L. Marcus WILHELMSSON[a*]

[a] *Department of Chemistry and Chemical Engineering, Chalmers University of Technology, Sweden.*
[b] *Department of Biology and Biological Engineering, Chalmers University of Technology, Sweden.*
[c] *Small Biosystems Lab, Condensed Matter Physics Department, Universitat de Barcelona, C/ Marti i Franques 1, 08028 Barcelona, Spain.*

** Corresponding authors,*

Marcus WILHELMSSON: marcus.wihelmsson@chalmers.se

Fredrik WESTERLUND: fredrik.westerlund@chalmers.se

Materials and Methods

DNA Hairpin Constructs

The DNA constructs used in this work are composed of 44 bases (20bp in the stem and 4 bases in the loop) inserted between two 29bp duplex handles and tCs[1] incorporated at various positions and numbers instead of C. The DNA hairpins were formed by hybridization of three different oligonucleotides (oligo 1, oligo 2 and splint as shown in Fig. S7 and Table S1). The oligonucleotides were synthesized and supplied by ATDBio, Southampton, UK and hybridized in-house. Oligo 1 contains the handle (29 bases) and a part of the DNA hairpin (14 bases) and is biotinylated at its 5' end. The remaining part of the DNA hairpin sequence (30 bases) along with the second handle (29 bases) comprises oligo 2. Terminal transferase reactions were performed using dUTP-digoxigenin (Digoxigenin-11-dUTP, Roche) to dATP in a ratio of 1:10 to modify the 3'end with a digoxigenin tail (Oligonucleotide Tailing Kit, Roche). Then, oligo 2 was purified using the Qiaquick Nucleotide Purification Kit (Qiagen). The dsDNA handles were obtained by hybridizing the splint (29 bases) to both oligo 1 and oligo 2 separately as follows: 95°C for 1 min, 80°C for 10 s, then decreased from 85°C to 40°C at 0.5°C per step with a hold time of 10 s per step. Then, oligo 1 and oligo 2 were mixed and allowed to hybridize with a decrease in temperature from 40°C to 10°C at 0.5°C with a hold time of 20 s at each step. Finally, the DNA hairpins were ligated using T4 DNA ligase (New England Biolabs) using the protocol: 16°C for 16 hours, then increased to 65°C for 10 min and decrease to 10°C. The assembled DNA hairpins were stored at -20°C and used for the experiments. DNA hairpins with handles tailed with biotin on the 5' end and digoxigenin on the 3' end allowed attachment to streptavidin and antidigoxigenin-coated polystyrene beads, respectively. Streptavidin beads with a diameter of 2.0-2.9 µm were purchased from Kisker Biotech and anti-digoxigenin beads were prepared in-house from Protein-G polystyrene beads with a diameter of 3.0-3.4 µm purchased from Kisker Biotech. 1ml of Protein G beads were washed twice with linking buffer (100mM Na_2HPO_4, 100mM NaCl, pH 8.5). Resuspended beads were incubated with 120 µl of anti-digoxigenin polyclonal antibodies (Roche) and 60 µl of cross-linker dimethyl pimelimidate (Thermo Fischer). After incubation, the anti-digoxigenin coated beads were washed twice with PBS buffer (140mM NaCl, 2.7mM KCl, 61mM K_2HPO_4, 39mM KH_2PO_4, 0.02% NaN_3, pH 7.0) and finally resuspended in 1 ml of PBS buffer.

Optical Tweezers and Force-Distance Experiments

The experiments are performed using an in-house built optical tweezers instrument described in detail in Bosaeus *et al.*[2] Two counter-propagating 150 mW, 845 diode lasers were used to form a single trap within the microfluidic chamber mounted on a motorized stage. The fluidic chamber is divided into three channels; two of them are used to dispense the two different types of coated beads and the third channel contains the trap and the micropipette. The optical trap is used to capture one of the polystyrene coated beads and the other coated bead is immobilized by suction at the tip of the micropipette. Optical fibers are used to guide the laser beams and the position of the trap is measured by redirecting 5% of the light intensity onto a position sensitive detector (PSD). The remaining light is focused through a water-immersion objective lens (60X, NA 1.20) to form the trap. The light exiting the trap is collected by an identical objective lens and redirected to a PSD and photodiode to measure the forces acting on the trapped bead in all three dimensions. A quarter-wave plates and polarizing beam splitters are used to redirect the light exiting the condensing objective lens to the force detector by turning the polarization of light 90° relative to the light entering the objective and monitored individually. The forces are measured based on the conservation of light momentum.[2-4]

The single-molecule mechanical (un)folding experiments were performed by attaching the biotinylated handle on the DNA hairpin to a streptavidin-coated bead and the digoxigenin handle to the anti-digoxigenin-coated bead. The streptavidin-coated bead was held on the micropipette and the anti-digoxigenin bead was trapped in the optical trap, as shown in Fig. 1B. A tether containing a single DNA hairpin was established between the two beads, the force-distance experiments were performed by moving the trap at a constant velocity (100 nm/s) in a specific force range to follow unfolding and folding of the hairpins recurrently. Typical force-distance curves are shown in Fig. 1C and Fig. S1 and (un)folding forces are observed as sudden jumps in force and extracted using custom-made MATLAB programs. Data were recorded at a frequency of 1 kHz. All the experiments were carried out at a constant temperature of 23.5±1°C. Measurements were performed in a buffer containing 10mM Tris pH 7.4, 1mM EDTA and using two different NaCl salt concentrations of (1M and 50mM).

Free-Energy Calculation

Single-molecule force measurements can be used to determine the free-energy difference (ΔG^0) between the folded and unfolded states. Crook fluctuations theorem (CFT)[5, 6] can be used to extract equilibrium free energies from non-equilibrium processes, such as mechanical (un)folding. The work done during unfolding and folding of the DNA hairpins was calculated by integrating the area below the force-distance curve (as shown in Fig. S4A), where the force transition occurs (λ_0 and λ_1) for each cycle. The calculated work done during unfolding (W) and recovered during folding (-W) was used to construct probability distributions. The crossing point of two work distributions is equal to the free energy differences (ΔG^0) as shown in Figure S4. The ΔG^0 measured contains contributions from the hairpin under study, the handles and the bead in the optical trap.[7, 8] The free energy of formation of the hairpin (ΔG) can be estimated using the following equation,

$$\Delta G^0 = \Delta G + \Delta W^{st} + \Delta W^{handles} + \Delta W^{bead} \quad (1)$$

The terms ΔW^{st} and $\Delta W^{handles}$ are the work needed to stretch the hairpin and the handles, respectively. ΔW^{bead} is the work needed to displace the bead in the optical trap. The work needed to stretch the molecule (ΔW^{st}) was obtained by subtracting the work needed to stretch the unfolded ssDNA using the inextensible worm-like chain (WLC) model[5] and the work needed to orient the hairpin. The hairpin is modelled as a freely jointed chain with a Kuhn and monomer length equal to the B-DNA hairpin diameter d=2.0 nm. The contribution from the handles ($\Delta W^{handles}$) and the beads (ΔW^{bead}) are combined using the measured effective stiffness, assuming it to be constant in the range of forces ($f_0<f<f_1$) studied.[5] The free energy of formation of the DNA hairpin (ΔG) was obtained using equation 1. The free energy calculations were done using custom-made MATLAB programs.

Thermal Melting and Circular Dichroism (CD) Measurements

Melting studies on the DNA hairpins (without handles) were performed using a Varian Cary 4000 spectrophotometer equipped with a programmable multicell temperature block. Hairpin concentrations were set to 2 µM using the absorbance at 260 nm in a buffer containing 10mM Tris pH 7.4, 1mM EDTA and two different NaCl salt concentrations of (5mM and 50mM). The absorption spectra were measured using a spectral bandwidth (SBW) of 2 nm and signal averaging time of 2 s. The samples were heated from 25°C to 95°C at a ramp rate of 1°C/min. The temperature was held at 95°C for 5 min and cooled to 5°C at the same rate. Absorption at 260 nm was measured with a temperature interval of 1°C for two consecutive cycles of heating and cooling (as shown in Fig. S2B). The melting temperatures (T_m) of the hairpins were determined as the maximum of the first derivative of the melting curves. The alpha curves (fraction of single strands in duplex state) were obtained by selecting the lower and upper linear baseline and calculation the fraction by normalizing fractional absorbance to the total absorbance change over entire temperature range.[9] The alpha curves were fitted with Boltzmann sigmoidal fitting function in the Origin software to check if the melting curves fit with a two-state model.

Circular dichroism (CD) spectra were recorded for the hairpins at a concentration of 6 µM at 20°C using a Chirascan CD spectrometer (Applied Photophysics). The spectra were recorded from 210 to 475 nm at a SBW of 1 nm and averaged over three scans and then background corrected using a blank sample. The scan rate was set to 0.2 s per point with a step size of 1 nm.

Estimation of Thermodynamic Parameters

Thermodynamic parameters like enthalpy (ΔH) and entropy (ΔS) can be computed by combining the thermal measurements and the mechanical measurements at the same salt concentration (50mM NaCl). The free energy of formation (ΔG) between the two states was calculated as:

$$\Delta G(T) = \Delta H - T\Delta S \qquad (2)$$

ΔG is the free energy of formation calculated using CFT from the single-molecule (un)folding measurements at 23.5±1°C (experimental temperature, T). The melting temperature (T_m) is the temperature at which the unfolded and the folded state exist at equal probability (no difference in energy between the two states). So, equation 1 can at T_m be written as,

$$\Delta H - T_m \Delta S = 0 \qquad (3)$$

The entropy is calculated by combing equation 2 and 3 and it is then used to calculate the enthalpy using equation 2 or 3.

$$\Delta S = \Delta G/(T_m - T) \qquad (4)$$

The enthalpies and entropies computed for the DNA hairpins (unmodified, 1-tC and 2-tC(stack) are shown in Table 2.

The enthalpy and entropy for the other hairpins (2-tC, 3-tC, Abasic, 2-tC(opp) can be estimated by computing ΔG at 50mM NaCl from ΔG at 1M NaCl values and combing with T_m from measurements at 50mM NaCl. The free energy of formation exhibits a simple linear logarithmic dependence with salt concentration[10] as given by:

$$\Delta G_0 = \Delta G - m\log(C) \qquad (5)$$

where ΔG_0 is the free energy at salt concentration C (50mM), ΔG is the free energy at the reference condition (1M), and m (kcal mol^{-1}) is a correction factor (0.11*number of bp). We assume that the contribution from the 4-nucleotide loop in the salt dependence to the overall free-energy of the structure is negligible, and we assume that the tC modifications have the same salt dependence as any of the other Watson-Crick bases. The entropy and enthalpy estimated after salt correction for the DNA hairpins are shown in Table S4.

Generalized Linear Mixed Models

Statistical significance of (un)folding forces obtained from unmodified and tC incorporated hairpins were analyzed using the generalized linear mixed model (GLMM) to mainly consider the random (stochastic) effects present in the experiments (Table S2 and Table S3). With a bead pair, multiple cycles of stretching and relaxing the hairpin were performed, yielding different (un)folding forces for each cycle. Subsequently, we change to different bead pairs and acquired additional data. The forces obtained within a set of bead pairs is dependent, whereas the forces between different sets of bead pairs are

independent. The experiments were neither completely independent nor dependent, so to account for the heterogenous data, we used GLMM and tested the significance using mixed function of the apex package in the R software.[11, 12]

Supplementary Figures and Tables

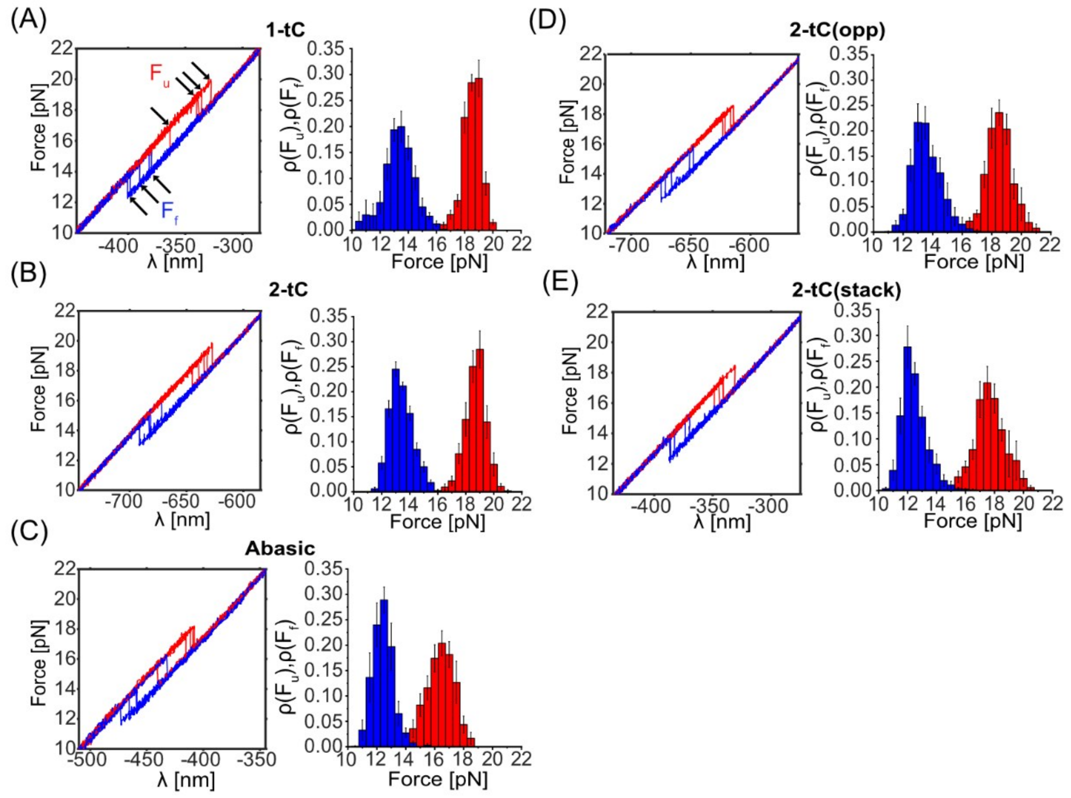

Fig. S1: Force-distance curves and rupture force histograms obtained from unfolding and folding of tC modified hairpins at 1M NaCl. (A) 1-tC (B) 2-tC (C) Abasic (D) 2-tC(opp) (E) 2-tC(stack). The other hairpins can be found in Fig. 1 of the main text (Unmodified, 3-tC).

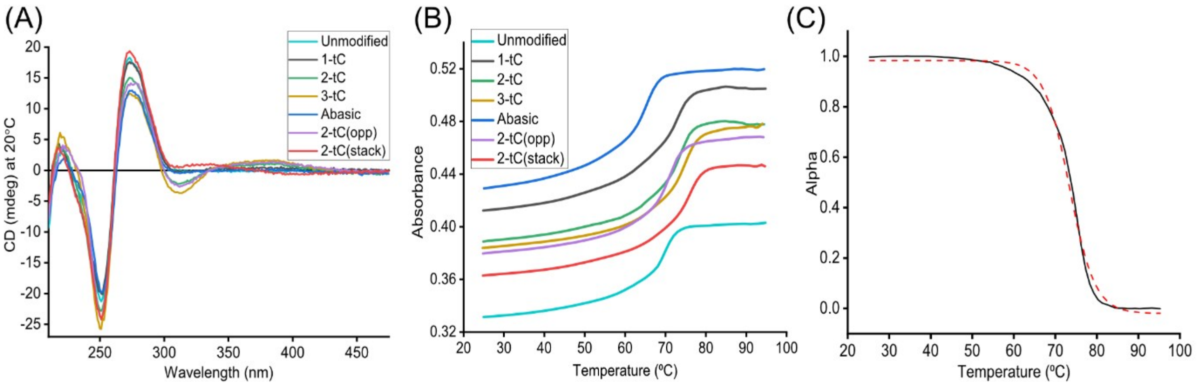

Fig. S2: Bulk measurements with tC-modified DNA hairpins. (A) CD spectra of the different DNA hairpins measured at 6 µM. (B) Thermal melting curves using the change in absorbance at 260 nm with changing temperature for the different DNA hairpins at 2 µM. Bulk measurements were performed in a Tris-based buffer with 5mM NaCl. (C) Fitting of alpha curve (black) with Boltzmann sigmoidal equation (red dashed; adjusted R-Square: 0.9963) for 3-tC melting curve.

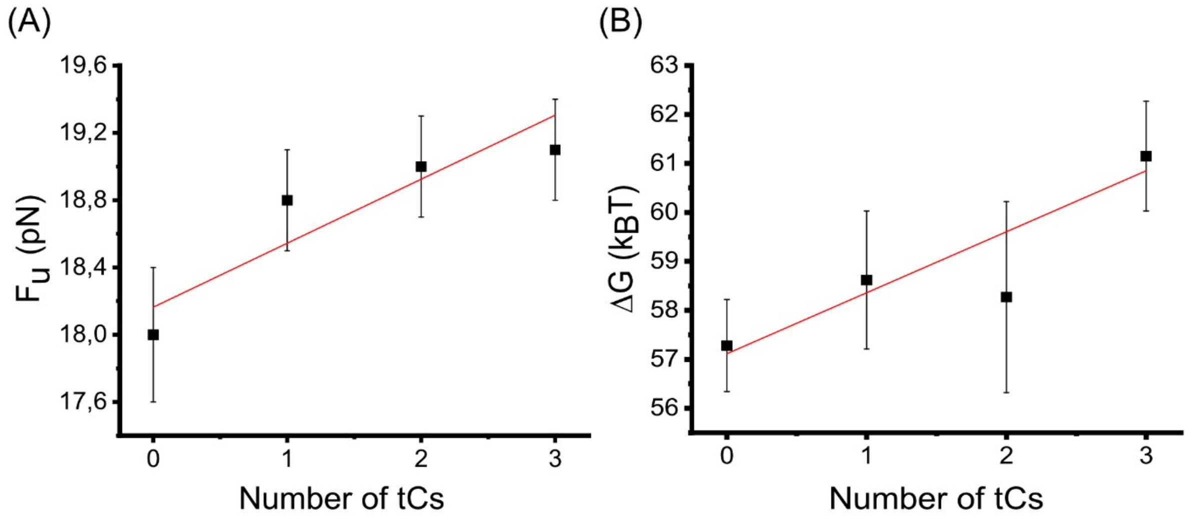

Fig. S3: Increasing trend in unfolding force (F_u), free energy (ΔG) and with the number of tCs in the DNA hairpins. (A) Linear fit of unfolding force data obtained from unmodified, 1-tC, 2-tC and 3-tC modified hairpins with Pearson's r: 0.91, linear fit r: 0.85. (B) Linear fit of free energy data obtained from unmodified, 1-tC, 2-tC and 3-tC modified hairpins with Pearson's r: 0.88, r: 0.82.

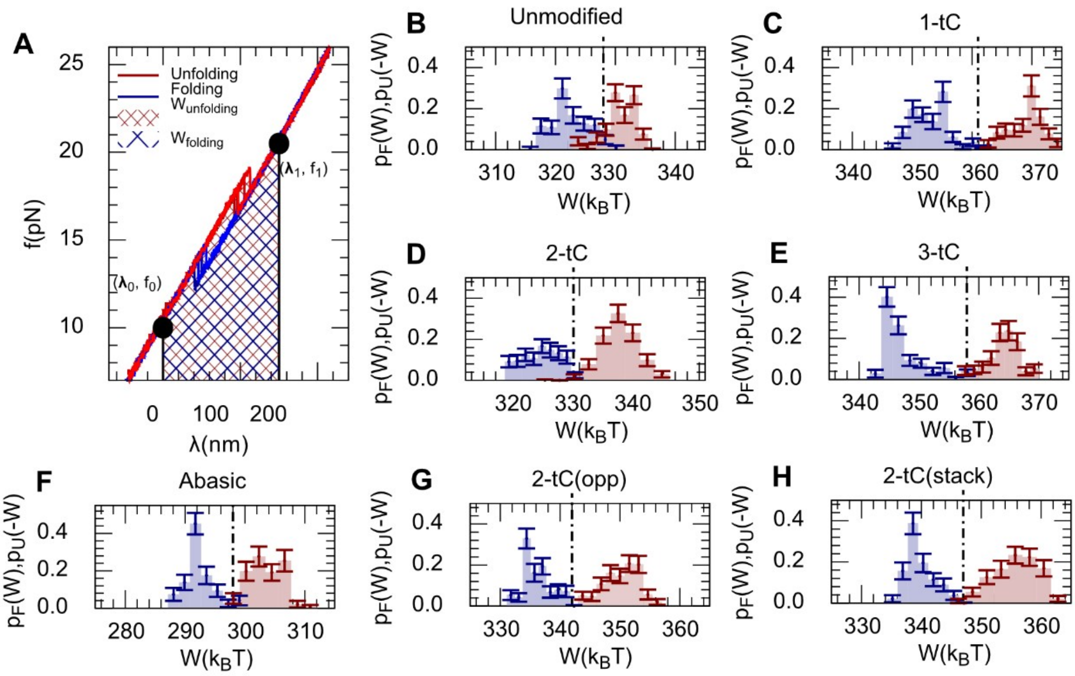

Fig. S4: Free-energy calculation. (A) Force-distance curves indicating the area used to calculate the work done during unfolding (red) and folding (blue) of the DNA hairpins. The work done is calculated by integrating the dashed areas. (B-H) Work probability distributions obtained from a single tether during unfolding (red) and folding (blue) for the different DNA hairpins. The crossing point of the two distributions (dashed line) indicates the free-energy differences (ΔG^0).

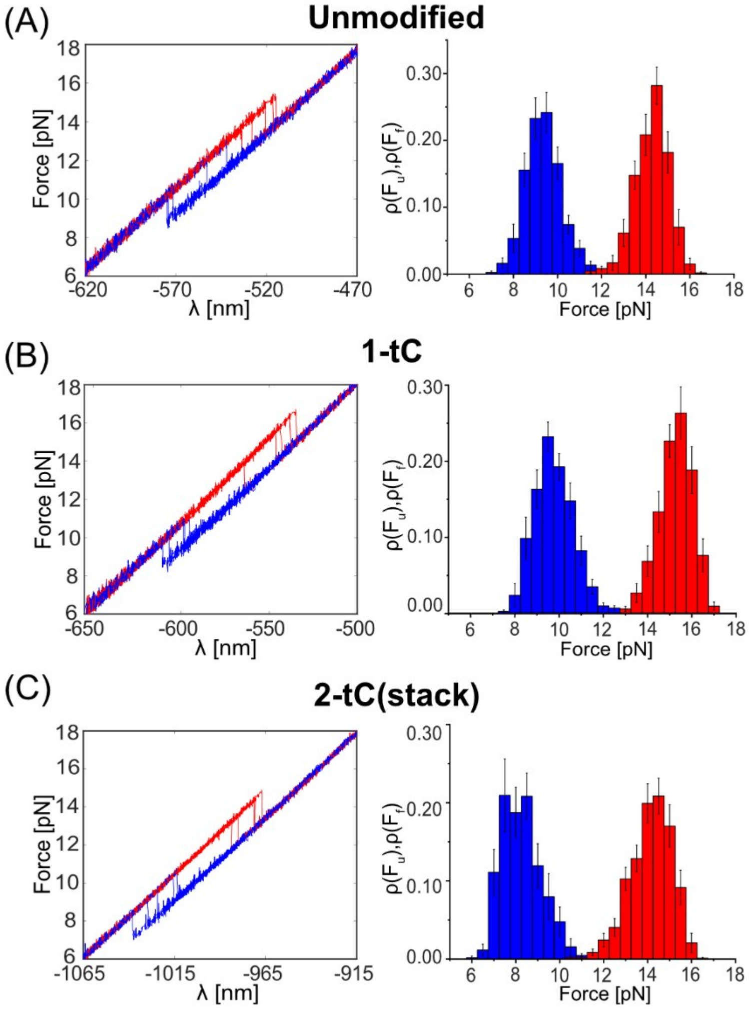

Fig. S5: Force-distance curves and force histograms obtained from unfolding (red) and folding (blue) of tC-modified hairpins at 50mM NaCl. (a) Unmodified, (b) 1-tC, (c) 2-tC(stack).

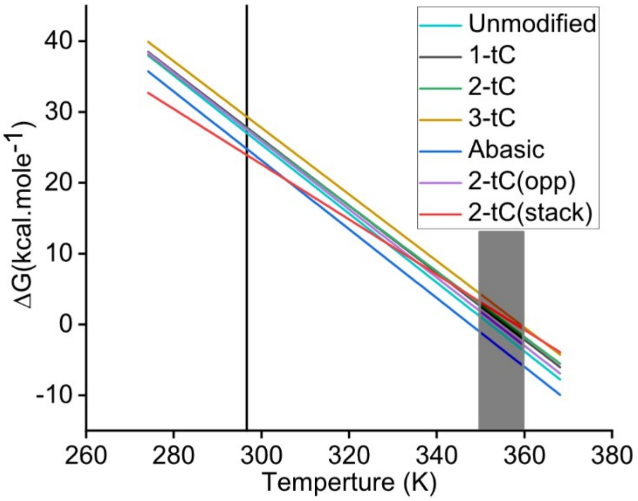

Fig. S6: Change in free energies for the DNA hairpins with temperature. Enthalpy and entropy are estimated after salt correction on ΔG from 1M NaCl to 50mM NaCl and T_m from 50mM NaCl as mentioned in Materials and Methods. Vertical line represents the temperature of optical tweezers and shaded area corresponds to melting temperature of hairpins.

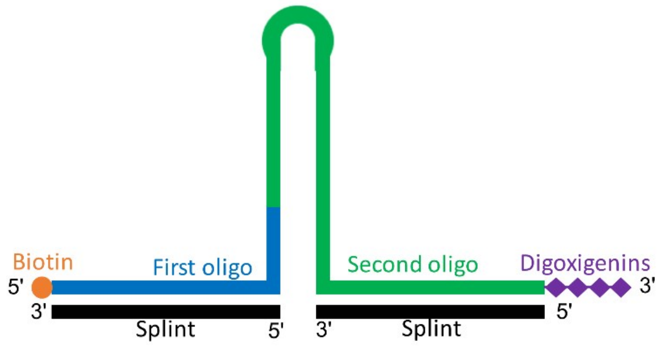

Fig. S7: Sketch of the synthesized DNA hairpins with the three different oligonucleotides.

Table S1: DNA oligonucleotides used. The DNA hairpin is constructed using hybridization of three oligonucleotides (oligo 1, oligo 2 and splint). The main part of the DNA hairpin (without handles) is shown in bold and the tC modifications in red.

Oligonucleotide	Sequence
Unmodified	Oligo 1: 5 -Biotin-AGTTAGTGGTGGAAACACAGTGCCAGCGC**GCGCCGCATCCTAG**-3’<br>Oligo 2-1
1-tC	Oligo 1: 5 -Biotin-AGTTAGTGGTGGAAACACAGTGCCAGCGC**GCGCCGCATCtCTAG**-3’<br>Oligo 2-1
2-tC	Oligo 1: 5 -Biotin-AGTTAGTGGTGGAAACACAGTGCCAGCGC**GCGCCGtCATCtCTAG**-3’<br>Oligo 2-1
3-tC	Oligo 1: 5 -Biotin-AGTTAGTGGTGGAAACACAGTGCCAGCGC**GtCGCCGtCATCtCTAG**-3’<br>Oligo 2-1
Abasic	Oligo 1: 5 -Biotin-AGTTAGTGGTGGAAACACAGTGCCAGCGC**GCGCCGCATCtCTAG**-3’<br>Oligo 2-2
2-tC(opp)	Oligo 1: 5 -Biotin-AGTTAGTGGTGGAAACACAGTGCCAGCGC**GCGCCGtCATCCTAG**-3’<br>Oligo 2-3
2-tC(stack)	Oligo 1: 5 -Biotin-AGTTAGTGGTGGAAACACAGTGCCAGCGC**GCGCCGCATtCtCTAG**-3’<br>Oligo 2-1
Oligo 2-1	5’-phosphorylated-**CATATTGAAAAATATGCTAGGATGCGGCGC**AGTTAGTGGTGGAAACACAGTGCCAGCGC-3’
Oligo 2-2	5’-phosphorliated-**CATATTGAAAAATATGCTAXGATGCGGCGC**AGTTAGTGGTGGAAACACAGTGCCAGCGC-3’
Oligo 2-3	5’-phosphorliated-**CATATTGAAAAATATGCTAGGATGtCGGCGC**AGTTAGTGGTGGAAACACAGTGCCAGCGC-3’
Splint	5’-GCGCTGGCACTGTGTTTCCACCACTAACT-3’

Table S2: Statistical significance for unfolding force (F_u) using GLMM. The unfolding force obtained from different DNA hairpin were compared to calculate the p-value using GLMM in R software. ***p≤0.001, **p≤0.01,*p≤0.05 and ns.p>0.05

DNA Hairpin	Unmodified	1-tC	2-tC	3-tC	Abasic	2-tC(opp)	2-tC(stack)
Unmodified		***	***	***	***	**	ns
1-tC			ns	**			
2-tC				ns			

Table S3: Statistical significance for folding force (F_f) using GLMM. The folding force obtained from different DNA hairpin were compared to calculate the p-value using GLMM in R software. ***p≤0.001, **p≤0.01,*p≤0.05 and ns.p>0.05

DNA Hairpin	Unmodified	1-tC	2-tC	3-tC	Abasic	2-tC(opp)	2-tC(stack)
Unmodified		ns	ns	ns	**	ns	**

Table S4: Salt correction and estimation of enthalpy and entropy. [a] Free energy calculated using CFT from force-distance experiments at 1M NaCl. [b] Free energy at 50mM NaCl computed from 1M NaCl using salt correction represented as mean±standard error of mean. [c] Melting temperatures (T_m, 50mM NaCl) reported as mean±standard deviation. [d] T_m for 3-tC was obtained from measurement using a different instrument (old instrument broken) in which the T_m for the unmodified was measured to be 80.6°C [e] Enthalpy and entropy calculated by combining ΔG_0 (50mM NaCl) and T_m (50mM NaCl) and reported as mean±standard error of mean. The values reported in the parentheses implies to the error associated with the last significant figure.

DNA Hairpins	ΔG (1M NaCl) [a] (k_BT)	ΔG_0 (50mM NaCl) [b] (k_BT)	T_m [c] (50mM NaCl) (°C)	-ΔH [e] (kcal mol^{-1})	-ΔS [e] (cal K^{-1} mol^{-1})
Unmodified	57 (1)	46 (1)	79.0 (5)	172 (4)	487 (14)
1-tC	59 (1)	47 (1)	82.2 (7)	168 (5)	474 (17)
2-tC	58 (2)	47 (2)	83.0 (5)	165 (6)	464 (21)
3-tC	61 (1)	50 (1)	85.9 (6)[d]	169 (4)	470 (16)
Abasic	53 (1)	42 (1)	74.5 (7)	169 (5)	486 (16)
2-tC(opp)	58 (1)	47 (1)	80.6 (6)	170 (5)	482 (16)
2-tC(stack)	52 (1)	41 (1)	84.9 (8)	140 (5)	390 (17)

Table S5: Salt correction and estimation of enthalpy and entropy. [a] Free energy calculated using CFT from force-distance experiments at 1M NaCl. [b] Free energy at 5mM NaCl computed from 1M NaCl using salt correction represented as mean±standard error of mean. [c] Melting temperatures (T_m, 5mM NaCl) reported as mean±standard deviation. [d] T_m for 3-tC was obtained from measurement using a different instrument (old instrument broken) in which the T_m for the unmodified was measured to be 71.2°C [e] Enthalpy and entropy calculated by combining ΔG_0 (5mM NaCl) and T_m (5mM NaCl) and reported as mean±standard error of mean. The values reported in the parentheses implies to the error associated with the last significant figure.

DNA Hairpins	ΔG (1M NaCl) [a] (k_BT)	ΔG_0 (5mM NaCl) [b] (k_BT)	T_m [c] (5mM NaCl) (°C)	-ΔH [e] (kcal mol^{-1})	-ΔS [e] (cal K^{-1} mol^{-1})
Unmodified	57 (1)	37 (1)	69.7 (5)	163 (5)	476 (17)
1-tC	59 (1)	39 (1)	72.2 (6)	160 (6)	462 (20)
2-tC	58 (2)	38 (2)	73.7 (7)	156 (8)	449 (25)
3-tC	61 (1)	41 (1)	76.4 (7)[d]	160 (5)	459 (17)
Abasic	53 (1)	34 (1)	65.0 (5)	161 (6)	475 (19)
2-tC(opp)	58 (1)	38 (1)	71.0 (5)	163 (6)	473 (19)
2-tC(stack)	52 (1)	32 (2)	75.8 (6)	126 (6)	361 (19)

References:

1. K. C. Engman, P. Sandin, S. Osborne, T. Brown, M. Billeter, P. Lincoln, B. Norden, B. Albinsson and L. M. Wilhelmsson, *Nucleic Acids Res*, 2004, 32, 5087-5095.
2. N. Bosaeus, A. H. El-Sagheer, T. Brown, B. Akerman and B. Norden, *Nucleic Acids Res*, 2014, 42, 8083-8091.
3. S. B. Smith, Y. Cui and C. Bustamante, *Methods Enzymol*, 2003, 361, 134-162.
4. S. B. Smith and C. Bustamante, *United States Patent no.* 7,133,133 B2. 2007.
5. A. Alemany and F. Ritort, *Biopolymers*, 2014, 101, 1193-1199.
6. G. E. Crooks, *J Stat Phys*, 1998, 90, 1481-1487.
7. D. Collin, F. Ritort, C. Jarzynski, S. B. Smith, I. Tinoco, Jr. and C. Bustamante, *Nature*, 2005, 437, 231-234.
8. A. Mossa, M. Manosas, N. Forns, J. M. Huguet and F. Ritort, *J Stat Mech-Theory E*, 2009, 2009, 1-28.
9. P. M. Vallone, T. M. Paner, J. Hilario, M. J. Lane, B. D. Faldasz and A. S. Benight, *Biopolymer*s, 1999, 50, 425-442.
10. J. M. Huguet, C. V. Bizarro, N. Forns, S. B. Smith, C. Bustamante and F. Ritort, *Proc Natl Acad Sci U S A*, 2010, 107, 15431-15436.
11. V. M. Laurent, A. Duperray, V. Sundar Rajan and C. Verdier, *PLoS One*, 2014, 9(5), 1-11.
12. V. Sundar Rajan, V. M. Laurent, C. Verdier and A. Duperray, *Biophys J*, 2017, 112, 1246-1257.
